# A Review of Precision Oncology Knowledgebases for Determining the Clinical Actionability of Genetic Variants

**DOI:** 10.3389/fcell.2020.00048

**Published:** 2020-02-11

**Authors:** Xuanyi Li, Jeremy L. Warner

**Affiliations:** ^1^Vanderbilt University School of Medicine, Nashville, TN, United States; ^2^Department of Medicine, Vanderbilt University, Nashville, TN, United States; ^3^Department of Biomedical Informatics, Vanderbilt University, Nashville, TN, United States

**Keywords:** precision oncology, knowledgebase, variant interpretation, actionability, targeted therapy, genotype-selective clinical trial, tumor genetic testing

## Abstract

The increased availability of tumor genetic testing and targeted cancer therapies contributes to the advancement of precision medicine in the field of oncology. Precision oncology knowledgebases provide a way of organizing clinically relevant genetic information in a way that is easily accessible for both oncologists and patients, facilitating the genetic-based clinical decision making. Many organizations and companies have built precision oncology knowledgebases, intended for multiple users. In general, these knowledgebases offer information on cancer-related genetic variants as well as their associated diagnostic, prognostic, and therapeutic implications, but they often differ in their information curations, designs, and user experiences. It is advisable that oncologists use multiple knowledgebases during their practice to have them complement each other. In the future, convergence toward common standards and formats is needed to ensure that the comprehensive knowledge across all sources can be unified to bring the oncology community closer to the achievement of the goal of precision oncology.

## Introduction

Precision oncology is defined as utilizing tumor molecular profiles to identify diagnostic, prognostic, and therapeutic implications pertaining to the specific tested cancer (Schwartzberg et al., 2017). Fundamentally, precision oncology is based upon the idea that tumor biomarkers are predictive of disease phenotype, clinical outcomes, and therapy responses. The first-ever cancer precision therapy under this definition was imatinib for chronic myeloid leukemia (Druker et al., 2001). Since then, a growing number of therapies targeted at specific genetic alterations have been introduced. Targeted therapies have led to improved outcomes in cancer patients with targetable mutations (Schwaederle et al., 2015, 2016). They have been shown to benefit patients with hard-to-treat cancer without increasing healthcare costs (Massard et al., 2017; Haslem et al., 2017).

**FIGURE 1:**
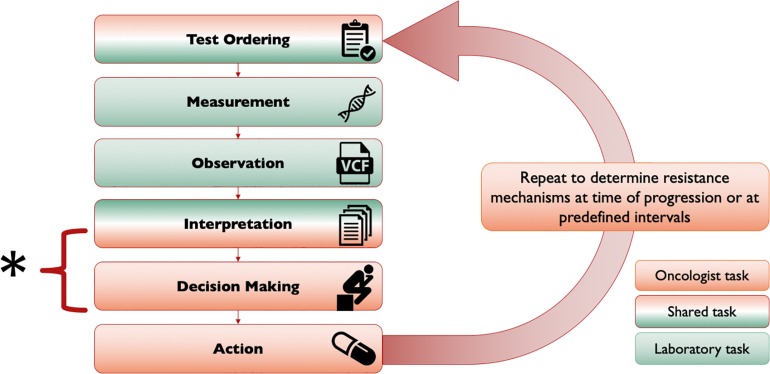
NGS workflow. The processes of ordering, measuring, interpreting, and acting upon clinical NGS information are complex and shared across clinician specialties. This schema outlines the tasks in the typical workflow of precision oncology decision making. Notably this process is iterative, with repetition typically triggered by progression or a prespecified time interval. The brackets illustrate the portion of the workflow that is the subject of this focused review. Traditionally, the test ordering and interpretation steps have clear hand-offs, although roles are becoming increasingly shared e.g., with the advent of molecular tumor boards. While the discussed knowledgebases may have purposes outside of this narrow scope, they also have clear utility in this part of the workflow. Conversely, resources such as COSMIC and dbSNP, which are useful for the laboratory professionals and pathologists’ role in interpreting sequencing data and annotating variants, are deliberately omitted in this mini-review, as they do not typically have a role in this portion of the workflow.

Tumor molecular profiles consist of DNA, RNA, and protein alterations, as well as epigenetic changes. Currently, the most widely used molecular profiling method is tumor DNA next-generation sequencing (NGS) (Tran et al., 2013; Van Allen et al., 2014). The introduction of NGS into clinical oncology has provided oncologists with a large amount of genomic information, which can be incorporated into clinical decision making. As shown in Figure 1, the tasks of ordering, measuring, interpreting, and acting upon NGS information are shared between laboratory professionals and pathologists and oncologists. Not all genomic information from NGS is clinically relevant. After genetic variants are identified through NGS, several additional steps are required and are typically performed by laboratory professionals and pathologists. “Noises” such as germline polymorphisms (Tuchman et al., 1997; Calado et al., 2008; Muller et al., 2010), false-positive artifacts generated by NGS technology, and clinically insignificant synonymous variants are filtered out from the report. Moreover, the clinical significance and actionability of the remaining variants will be assessed by laboratory professionals and pathologists in order to identify potential treatment targets (Gao et al., 2019). This complicated process causes difficulties in the implementation of precision cancer medicine in the clinic, which is additionally hampered by incomplete incorporation into clinical health information systems (Warner et al., 2016). Clinical evidence on tumor molecular biomarkers can be scattered and chaotic. With the introduction of large numbers of targeted therapy drugs and genotype-selected clinical trials, it is challenging for oncologists to fully explore all relevant treatment options.

For this purpose, there are several precision oncology knowledgebases developed at least in part for providing clinical decision support for oncologists in interpreting genomic data and identifying therapy targets (Bates et al., 2003). They can help oncologists determine therapeutic options and clinical trial availability. With few exceptions, the bulk of the content of these knowledgebases is based upon curation of published literature. These knowledgebases were created with slightly different goals in mind and differ in data curation approaches, information organization, and anticipated user experiences. The goal of this focused review is to introduce the knowledgebases specific to the tasks of interpretation and clinical decision making highlighted in Figure 1.

## Publicly Available Precision Oncology Knowledgebases

In response to the increasing demand for assistance with utilizing NGS information, several institutions have developed dedicated services e.g., the precision oncology decision support team at MD Anderson (Johnson et al., 2017) and the precision cancer medicine team at the Dana-Farber Cancer Institute (2019). Similarly, an increasing number of institutions are implementing molecular tumor boards. For oncologists who do not have access to such support, they may elect to use the publicly available precision oncology knowledgebases (Table 1), which allow users to access at least part of their curated content without charge. In alphabetical order:

**TABLE 1:** Summary of publicly available precision oncology knowledgebases.

Knowledgebase	Variant annotation	Drug availability	Trial matching	Literature citation	Website
CGI	Yes	Yes	No	Yes	www.cancergenomeinterpreter.org/
CIViC	Yes	Yes	No	Yes	https://civicdb.org
DEPO	Yes	Yes	No	Yes	http://depo-dinglab.ddns.net/
HemOnc.org	Yes	Yes	No	Yes	www.hemonc.org/
JAX CKB^+^	Yes	Yes	Yes*	Yes	https://ckb.jax.org/
MCG	Yes	Yes	Yes	No	https://www.mycancergenome.org/
OncoKB	Yes	Yes	No	Yes	https://oncokb.org/
PCT	Yes	Yes	Yes	Yes	https://pct.mdanderson.org/
PMKB	Yes	Yes	No	Yes	https://pmkb.weill.cornell.edu/
**Drug-associated knowledgebases**					
Genomics of drug sensitivity in cancer	Yes	Yes	No	No	https://www.cancerrxgene.org/
PharmGKB	Yes	Yes	No	No	https://www.pharmgkb.org/
Therapeutic target database	Yes	Yes	No	Yes	http://db.idrblab.net/ttd/

* Available only with subscription to CKB BOOST. ^+^Partially publicly available databases.

### Cancer Genome Interpreter (CGI)

Cancer Genome Interpreter (CGI) is a knowledgebase designed for the streamlining and automatization of the entire process of interpretation of cancer genomes (Tamborero et al., 2018). It incorporates several different databases for the annotation of alterations, the identification of driver mutations, the determination of variant actionability, and exploration of biomarker interactions. CGI is notable for its attempt to assess the tumorigenic potential of variants of unknown significance (VUS). VUS represents one of the challenges of interpretation of genomic information and significantly contributes to uncertainty in clinical decision making (Richter et al., 2013). CGI assesses for the tumorigenic potential of VUS via OncodriveMUT. OncodriveMUT employs a rule-based approach that combines features of the VUS, including the action of the gene, the consequence of the mutation, its position within the transcript, its prevalence within the human population, and whether the mutation occurs in a domain of the protein that is depleted of germline variants. CGI is hosted at the Barcelona Biomedical Genomics Lab.

### Clinical Interpretations of Variants in Cancer (CIViC)

Clinical Interpretations of Variants in Cancer (CIViC) is a crowdsourced knowledgebase for the clinical implications of cancer genome variants (Griffith et al., 2017). CIViC contains 6308 curated clinical evidence records for 2278 variants affecting 396 genes. Each evidence record is associated with a specific gene variant and contains information related to therapy, prognostics, diagnostics, or predisposition for cancer.

Clinical Interpretations of Variants in Cancer reports an evidence level ranging from established clinical utility (level A) to inferential (level E) for each evidence record it reports (Griffith et al., 2017). Also, CIViC’s open model can potentially facilitate the standardization of clinical variant interpretation but can also make it challenging to quality control the curated evidence. CIViC is hosted at the Washington University in St. Louis School of Medicine.

### Database of Evidence for Precision Oncology

Database of evidence for precision oncology (DEPO) is a curated knowledgebase of clinically relevant genomic variants and their respective drug therapies (Sun et al., 2018). It has a web portal that allows users to query the knowledgebase using standard gene names and optionally, cancer type, variant type or position. Notably, besides incorporating the present variant actionability information, DEPO also employs HotSpot3D (Niu et al., 2016), which analyzes the clustering of mutations on a three-dimensional protein structural model for predicting potential treatment targets. Also, DEPO utilizes the JSmol 3D molecular viewer to visualize the 3D mutational clusters and potentially druggable sites. DEPO is hosted by the Ding lab at Washington University in St Louis.

### HemOnc.org

HemOnc.org is a clinical knowledgebase for hematology and oncology providers that includes information on cancer drugs and regimens (JW is the Deputy Editor of HemOnc.org and co-founder of HemOnc.org LLC). Although HemOnc.org is not specifically a precision oncology knowledgebase, approximately 8% of its content is organized by genomic alterations. For example, non-small cell lung cancer (NSCLC) regimens are organized by EGFR, ALK, BRAF, and ROS1 mutation status; kinase inhibitors are categorized by their target kinase(s) and biomarker-specific FDA labeling is noted. These concepts and relationships are modeled in the derivative publicly available HemOnc vocabulary (Warner et al., 2019). Near-term efforts include structuring the genomic eligibility criteria for genomically defined regimens at the gene and variant levels. HemOnc.org is hosted by HemOnc.org LLC.

### Jackson Laboratory Clinical Knowledge Base (JAX CKB)

Jackson Laboratory Clinical Knowledge Base (JAX CKB) is a knowledgebase that incorporates integrated data related to cancer-associated genomic variants, therapeutic efficacy, and clinical trials for interpretation of genomic data in cancer (Patterson et al., 2016). The public access version of CKB contains information on 82 commonly known driver genes. On the JAX CKB website, users can search by gene, gene variants, drug, drug class, indication, and clinical trials. Notably, JAX CKB can build complex molecular profiles that allow for the association of therapeutic efficacy to multiple genetic alterations simultaneously instead of each variant in isolation (Patterson et al., 2019). JAX CKB is hosted at The Jackson Laboratory.

### My Cancer Genome (MCG)

My Cancer Genome (MCG) marks one of the earliest attempts at creating an online knowledge resource describing the clinical actionability of tumor molecular biomarkers (Taylor et al., 2016). The MCG website offers information on targeted therapy and clinical trials for an array of genetic variants that are involved in signaling pathways affected by different types of cancers. At the time of writing, MCG contains 2344 molecular biomarkers, including genetic biomarkers, protein expression markers, chromosomal markers, and markers of genomic instability. For each biomarker, MCG has information on the related disease, available drugs, as well as information on clinical trials that are currently recruiting.

Unlike many other precision oncology knowledgebases, MCG organizes clinical evidence for genomic variants in a disease-centric approach instead of a gene-centric approach (Gao et al., 2019). MCG distinguishes between genetic variants within the same gene (Swanton, 2012), which could potentially discourage the use of targeted agents outside their indicated genetic variants. MCG has a user-friendly web interface and is beneficial for patient education and empowerment (Kusnoor et al., 2016). Additionally, MCG uses a pathway approach to curate genetic variants, which supports pathway-based treatment strategies with vertical and parallel inhibition. A limitation of MCG is that clinical evidence is not prioritized based on a predefined schema for levels of evidence. MCG is hosted at Vanderbilt University Medical Center and has a commercial relationship with GenomOncology LLC (Cleveland, OH, United States).

### Personalized Cancer Therapy (PCT)

Personalized Cancer Therapy (PCT) is a knowledgebase for clinical evidence of tumor genomic variants (Kurnit et al., 2017; Dumbrava and Meric-Bernstam, 2018). At the time of writing, PCT provides publicly accessible information for 32 actionable genes. For each gene, PCT includes general gene information, variants in that gene, frequencies and outcomes of these variants, therapeutic implications of variants, FDA-approved drugs or investigational therapeutics in clinical trials, as well as information on related genotype-selected and -relevant clinical trials.

Unlike MCG, PCT is organized in a gene-centric and patient tumor type-agnostic fashion (Gao et al., 2019). This organizational approach is designed to facilitate the process of identifying available targeted therapies during a patient encounter. Notably, a substantial portion of this knowledgebase is not available to the public and is instead used only for in-house precision oncology decision support. PCT is hosted at the MD Anderson Cancer Center Sheikh Khalifa Bin Zayed Al Nahyan Institute for Personalized Cancer Therapy.

### Precision Medicine Knowledge Base (PMKB)

Precision Medicine Knowledge Base (PMKB) is an online knowledgebase for structured clinical-grade cancer mutation interpretations (Huang et al., 2017). At the time of writing, PMKB contains 2246 variants with 1767 graded interpretations. Similar to CIViC, PMKB rates variant interpretations by a numeric tier indicating the clinically actionability with tier 1 as strong evidence of clinical utility, tier 2 as potential clinical relevance, and tier 3 as undetermined clinical significance (Huang et al., 2017). The majority of variant interpretations in PMKB are rated as tier 2. Also, PMKB supports crowdsourcing of content by contributors in specific subspecialties. PMKB is hosted at Weill Cornell Medicine Englander Institute for Precision Medicine.

### Precision Oncology Knowledge Base (OncoKB)

Precision Oncology Knowledge Base (OncoKB) is a precision oncology knowledgebase of tumor markers and their related FDA-approved therapies and investigational agents that are under evaluation in clinical trials (Chakravarty et al., 2017). OncoKB offers information for about 5000 genetic variants in 642 cancer-associated genes from 45 tumor types. OncoKB is also tightly integrated into the local environment of the Memorial Sloan Kettering Cancer Center and can take information directly from the MSK-IMPACT testing to produce a nearly automated report (Schram et al., 2017; Zehir et al., 2017).

OncoKB highlights adverse outcomes of off-label use of targeted therapies in specific mutational contexts (Gao et al., 2019). Off-label use of cancer drugs is prevalent, especially in patients with rare cancers, for which randomized clinical trials may not be feasible (Conti et al., 2013). However, off-label medication use can be costly and can lead to adverse effects and compromise clinical trials (Krzyzanowska, 2013). A limitation of OncoKB is that the website does not contain information on genotype-selective clinical trials. OncoKB is hosted at the Memorial Sloan Kettering Cancer Center.

### Drug-Associated Knowledgebases

Several drug-associated knowledgebases also provide information on variant drugability. The therapeutic target database (TTD) provides information about the therapeutic targets and corresponding approved and experimental drugs (Liu et al., 2011). Its data can be explored for target and drug searches and the development of *in silico* target discovery tools. The Genomics of Drug Sensitivity in Cancer (GDSC) contains information on drug sensitivity in cancer cells and molecular markers of drug response (Yang et al., 2013). Notably, GDSC contains large scale genomic and cell-line anticancer drug sensitivity datasets that could facilitate the discovery of new therapeutic biomarkers. The Pharmacogenomics Knowledge Base (PharmGKB) is a public repository of information relevant to pharmacogenetics and pharmacogenomics (Klein and Altman, 2004; Whirl-Carrillo et al., 2012). It provides clinically relevant information, including dosing guidelines, annotated drug labels, and potentially actionable gene-drug associations and genotype-phenotype relationships.

## Commercially Available Knowledgebases

Besides those above publicly available precision oncology knowledgebases, there also exist several commercially available knowledgebases for interpreting cancer molecular information (Table 2). Several companies offer variant interpretation functionality that is bundled with their sequencing panel workflow (Zeng et al., 2019). Their reports may vary on whether variant annotations or literature citations are offered, but most are consistently reporting available targeted therapies and clinical trial matching.

**TABLE 2:** Summary of commercially available precision oncology knowledgebases.

Knowledgebases	Variant annotation	Drug availability	Trial matching	Literature citation
**Laboratory-based knowledgebases**				
Caris molecular intelligence	Yes	Yes	Yes	No
FoundationOne	Yes	Yes	Yes	Yes
Guardant360	No	Yes	Yes	No
NeoTYPE	Yes	Yes	Yes	No
Perthera	No	Yes	Yes	No
StrataNGS	No	Yes	Yes	No
Tempus	No	Yes	Yes	Yes
**Stand-alone knowledgebases**				
BaseSpace knowledge network	Yes	Yes	Yes	Yes
MolecularMatch	Yes	Yes	Yes	Yes
Oncomine knowledge-based reporter	Yes	Yes	Yes	Yes
QIAGEN clinical insight interpret	Yes	Yes	Yes	Yes
Watson for genomics	Yes	Yes	Yes	Yes

*This is a sample of the more widely known sources; omission is not intentional.*

There are also commercially available stand-alone variant interpretation tools. Their curated efforts are somewhat similar to the publicly available knowledgebases, but less is known about their data curation process. A recent study has been performed to compare some of these commercially available knowledgebases, which shows that they agree upon clinically relevant variants but have a relatively low overall concordance of evidence levels (Sakai et al., 2019).

## Prediction-Based Approaches

Several recent efforts have moved away from literature curation and toward the automated prediction of whether an observed somatic mutation is a driver of functional significance. These efforts are based on a broad array of bioinformatics pipelines and have succeeded in identifying functional mutations at scale (Bailey et al., 2018; Bertrand et al., 2018). However, there are studies showing that prediction models may have low specificity (Flanagan et al., 2010; Ernst et al., 2018). With the exception of CGI described above, they are out of scope for this review, which is primarily concerned with literature-based knowledge.

## Harmonization

With the large number of public and private knowledgebases now available, they will inevitably have differing content coverage and are likely to have discrepancies and disagreements. In recognition of this increasingly problematic situation, the Global Alliance for Genomics and Health (GA4GH) founded the Variant Interpretation for Cancer Consortium (VICC) as a Driver Project. One of the primary goals of the VICC is knowledgebase integration, through the creation of a meta-knowledgebase. This effort concatenated the results of six knowledgebases: CGI (2.7), CIViC (2.4), JAX CKB (2.6), MolecularMatch (commercial; not described in detail), OncoKB (2.3), and PMKB (2.5). This integration has resulted in 12,856 aggregate interpretations covering 3,437 unique variants in 415 genes, 357 diseases, and 791 drugs (Wagner et al., 2018).

## Discussion

Precision oncology knowledgebases offer potential solutions to many challenges associated with the implementation of an individualized treatment approach in clinical oncology (Andre et al., 2014). At their best, they are user-friendly repositories that organize complex information into easy-to-digest clinical evidence that can be applied to clinical decision making in real-time (Kusnoor et al., 2016; Dumbrava and Meric-Bernstam, 2018). They can also facilitate patient communication, which empowers patients to take an active role in their cancer treatment (Kusnoor et al., 2016).

On the other hand, there are several challenges associated with knowledgebases. Currently, few precision oncology knowledgebases are integrated into clinical workflows, making it difficult to assess user experience. There is also the issue with accountability and trustworthiness of the assertions made in the knowledgebases, which is important since they may affect clinical decisions. Most knowledgebases have extensive disclaimers and exculpatory clauses. However, it is unknown whether there would be legal liability in the case of malpractice lawsuits brought against a medical decision that directly involved the use of knowledgebases. Although there is no current FDA guidance for the clinical validity of somatic knowledgebases, it is possible that guidance similar to that for germline databases will be issued in the future (U.S. FDA, 2018). Precision oncology knowledgebases do not replace the experience and knowledge of a clinician, nor are they meant to replace a careful read of the primary literature. With each knowledgebase having its own profile of strengths and limitations, it is advisable that clinicians use multiple knowledgebases during their practice to take advantage of complementary information (Hughes et al., 2017). We have explored enabling such an approach with an app called SMART Cancer Navigator (Warner et al., 2018), which collects information from many knowledgebases for presentation to a clinical user, using application programming interfaces.

Cancer treatment is becoming more personalized. Although not addressed here, this includes integration of germline and family history information, which has been typically ignored in somatic NGS interpretations. For certain patient populations, e.g., children, the incidence of finding germline pathogenic variants causing hereditary cancer predisposition syndromes is quite high (Zhang et al., 2015). The major challenge as tumors are increasingly molecularly characterized is that literature support for any given mutation and especially for combinations of mutations and other factors such as germline will decrease. A potential solution is to rely on case reports of precision oncology; however, case reports are notoriously difficult to publish, are generally undervalued in the academic setting, and are unlikely to capture the true breadth of the clinical spectrum. Instead, there are several efforts intended to gather precision oncology diagnoses and outcomes at scale; many of these efforts are commercial e.g., (Singal et al., 2019). One public effort to create such a shared knowledgebase is the AACR Project GENIE, which is a multi-disciplinary effort that integrates de-identified clinical-grade cancer genomic data with clinical outcome data for tens of thousands of cancer patients treated at multiple institutions worldwide (AACR Project Genie Consortium, 2017). The GENIE database (JW has grant support from GENIE) has been used to train machine-learning classifiers and is in the process of adding more in-depth phenotype information (Nicora et al., 2019). In the future, the international oncology community should continue to converge on common standards for knowledge representation, so that a comprehensive and unified knowledgebase that links tumor molecular profile data with approved therapies and available clinical trials, which would lead to the achievement of the goal of precision oncology, can be achieved.

## Author Contributions

XL and JW made substantial contributions to the conception and design of the work, drafted and revised the work, approved the final submitted version, and agreed to be accountable for all aspects of the work in ensuring that questions related to the accuracy or integrity of any part of the work are appropriately investigated and resolved.

## Conflict of Interest

JW is the Deputy Editor of HemOnc.org and co-founder of HemOnc.org LLC; these positions are uncompensated and company shares have no monetary value. JW is a member of the IBM Watson Health Oncology and Genomics Advisory Council. JW receives grant funding from the National Institutes of Health, which also funds several of the knowledge bases mentioned; and AACR Project GENIE. The remaining author declares that the research was conducted in the absence of any commercial or financial relationships that could be construed as a potential conflict of interest.
